# Peer Review of “Predicting Escalation of Care for Childhood Pneumonia Using Machine Learning: Retrospective Analysis and Model Development”

**DOI:** 10.2196/71369

**Published:** 2025-03-04

**Authors:** Anonymous

**Keywords:** childhood pneumonia, community-acquired pneumonia, machine learning, clinical decision support system, prognostic care decision

*This is the peer-review report for “Predicting Escalation of Care for Childhood Pneumonia Using Machine Learning: Retrospective Analysis and Model Development.”*

## Round 1 Review

### General Comments

This paper [1] developed a machine learning approach that could predict community-acquired pneumonia prognosis, which is scaled into two levels, severe or nonsevere, and identify important clinical indices, such as hypoxia, respiratory distress, age, *z* score of weight for age, and antibiotic usage before admission. The machine learning–based clinical decision support system tool for childhood pneumonia could provide prognostic support for case management.

### Specific Comments

#### Major Comments

1. To enhance the manuscript’s grounding in current research and to provide a comprehensive context for the study, the authors are recommended to incorporate an evaluation of related literature in the Introduction and Discussion sections. This could include, but not be limited to, the following studies:

- Liu YC, Cheng HY, Chang TH, et al. Evaluation of the need for intensive care in children with pneumonia: machine learning approach. JMIR Med Inform. Jan 27, 2022;10(1):e28934. [doi: 10.2196/28934] [Medline: 35084358]
- Smith JC, Spann A, McCoy AB, et al. Natural language processing and machine learning to enable clinical decision support for treatment of pediatric pneumonia. AMIA Annu Symp Proc. Jan 25, 2020;2020:1130-1139. [Medline: 33936489]
- Kanwal K, Khalid SG, Asif M, Zafar F, Qurashi AG. Diagnosis of community-acquired pneumonia in children using photoplethysmography and machine learning-based classifier. Biomed Signal Process Control. Jan 2024;87:105367. [doi: 10.1016/j.bspc.2023.105367]
- Chang TH, Liu YC, Lin SR, et al. Clinical characteristics of hospitalized children with community-acquired pneumonia and respiratory infections: Using machine learning approaches to support pathogen prediction at admission. J Microbiol Immunol Infect. Aug 2023;56(4):772-781. [doi: 10.1016/j.jmii.2023.04.011] [Medline: 37246060]

The readers could have a more comprehensive understanding if the authors could include a concise evaluation of the prior literature in the current manuscript.

2. Considering the high stakes involved in pediatric care, particularly in intensive settings, it is critical to exam the false negative cases from the confusion matrices. Analyzing these cases for any common feature characteristics could provide insights into potential improvements in the predictive algorithm. This analysis should be clearly presented and discussed in the manuscript, emphasizing its importance in clinical decision-making.

3. The manuscript would benefit from a more detailed description of the cohort used in the study. Information on age, gender, and other clinical indices across the two groups (severe and nonsevere) would enable a better understanding of the study population. Additionally, providing the number of cases in each group would clarify the scope and scale of the study findings.

4. A detailed description of the data collection process is crucial for assessing the study’s applicability in real-world clinical settings. The manuscript should explicitly state the following:

- How and when clinical data, including features such as hypoxia and respiratory distress, were collected (eg, at the time of admission? or within 24 hours of admission?);
- The time frame considered for “antibiotic usage before admission” as relevant to the prediction model: This information is essential for replicability and for future applications of the findings in clinical workflows.

## Round 2 Review

I thank the authors for revising the manuscript.
